# Editorial: Research in: coping strategies

**DOI:** 10.3389/fpsyg.2023.1332218

**Published:** 2024-01-08

**Authors:** Ralph Erich Schmidt, Heng Choon (Oliver) Chan, Fabrizio Stasolla

**Affiliations:** ^1^Department of Psychology, University of Geneva, Geneva, Switzerland; ^2^Department of Psychiatry, Psychotherapy, and Psychosomatics, Psychiatric University Hospital Zurich, University of Zurich, Zurich, Switzerland; ^3^Department of Social Policy, Sociology, and Criminology, University of Birmingham, Birmingham, United Kingdom; ^4^Giustino Fortunato University, Benevento, Italy

**Keywords:** cognitive closure, coping, Coping Circumplex Model (CCM), emotion regulation flexibility, expectation violation, Model of Coping Modes (MCM), stress, ViolEx model

A vast body of research shows that stress may entail a whole range of negative consequences for physical and mental health (for a review, see O'Connor et al., 2021). However, the processes that lead from stress exposure to negative health outcomes are still not fully understood. According to one of the most influential accounts in the coping literature, Lazarus and Folkman's (1987) transactional model, the stress response is embedded in the person-environment interaction and largely determined by individual appraisal processes. When confronted with a stressful situation, the individual will evaluate the relevance of the stressors (primary appraisal) and its resources to deal with the situation (secondary appraisal). As a function of these appraisals, the individual will then select specific coping strategies. The ways in which the individual copes with the situation will not only shape the immediate stress response, but also long-term psychological wellbeing, life satisfaction, physical and mental health, as well as social functioning.

The four articles that are united in this Research Topic highlight currently active areas of coping research. The study of Exner et al. was inspired by the Model of Coping Modes (MCM) proposed by Krohne (1993). Following the MCM, individual differences in dispositional stress coping may be mapped on two main dimensions, namely cognitive avoidance and vigilance. Cognitive avoidance comprises a cluster of coping strategies that seek to shield from stimuli that might evoke emotional arousal and that are preferentially used by individuals who show an intolerance for emotional arousal. Vigilance, on the other hand, comprises a cluster of coping strategies that seek to reduce uncertainty resulting from unpredictability and that are preferentially used by individuals who show an intolerance for uncertainty. The combination of these two dimensions yields a classification of four main dispositional coping modalities: repressors (high cognitive avoidance, low vigilance); sensitizers (low cognitive avoidance, high vigilance); fluctuating coping (high on both dimensions); and non-defensive coping (low on both dimensions). In Exner et al.'s study, 96 university students in Germany were exposed to a stress induction (Mannheim Multicomponent Stress Test) and subsequently received a relaxation intervention while subjective ratings of stress and relaxation as well as objective measures of heart rate, heart rate variability and blood pressure were collected. The main findings were that repressors reported lower subjective stress levels and higher levels of relaxation throughout the two phases, whereas fluctuating copers showed lower heart rate and higher heart rate variability when compared with non-defensive copers. According to the authors of the study, the novel finding regarding fluctuating copers could suggest that these individuals, who score high both on cognitive avoidance and vigilance, might have had to learn a wide variety of coping strategies in their lives and that they flexibly adapt the use of strategies to situational characteristics. In contrast, non-defensive copers, who are able to tolerate both emotional arousal and uncertainty, might have developed a more restricted repertoire of coping strategies. This interpretation is in accord with other findings indicating situationally adaptive use of strategies in fluctuating copers (Schmukle et al., 2000) and more generally with the literature of emotion regulation flexibility (Nardelli, 2023).

The study by Henss and Pinquart investigated how 297 university students in Germany coped with positive and negative expectation violations in an anagram test that was made relevant to their academic self-concept. Following the VioLex model (*Viol*ated *Ex*pectations), they postulated that the students might use cognitive strategies of expectation change (accommodation), cognitive strategies of minimizing the impact of expectation violations (immunization), or behavioral strategies that increase the probability of expectation confirmation and decrease the probability of expectation violation (Gollwitzer et al., 2018). The strategies of accommodation and immunization mirror the previously mentioned strategies of vigilance and cognitive avoidance, respectively. Henss and Pinquart also included a measure of cognitive closure (for this concept, see Webster and Kruglanski, 1994) in their design and surmised that students scoring high on this dimension might be particularly prone to reducing uncertainty following expectation violations by engaging in accommodation and assimilation. The main findings were that students tended to accommodate and assimilate more strongly after negative expectation violations when compared with positive expectation violations and that cognitive closure did indeed fuel accommodation and assimilation following worse-than-expected performance.

The remaining studies of this Research Topic cast a light on two other important aspects of coping, specifically the development of coping skills and the role of social support. Arthur et al. analyzed the effects of a 10 × 40 min emotion regulation intervention in a sample of 73 children aged 5–6 in Australia. The authors found that parents whose children received the intervention reported a significant improvement in their children's anger coping 2 months later. In terms of early stress prevention, the findings of this pilot study are highly promising. Based on an online survey with 221 shadow education industry tutors in China, Ji et al. found that the association between dispositional hope and life satisfaction was partly mediated by the use of positive stress coping strategies that, in turn, increase perceived social support. This finding highlights the social ramifications of individual coping styles.

A challenge for future research will be to develop theoretical models that capture the complex interplay between physiological, cognitive, affective, behavioral, and social aspects of stress coping, ideally in a perspective of developmental psychopathology. The recently proposed Coping Circumplex Model (CCM) by Stanisławski (2019) illustrates the interest of theoretical integration. The CCM posits that individuals under stress face two main tasks: they need to solve the problem and to regulate their emotions. The CCM represents these two dimensions in a circular space that comprises eight coping strategies: positive emotional coping, efficiency, problem solving, preoccupation with the problem, negative emotional coping, helplessness, problem avoidance, and hedonic disengagement. Social forms of coping may easily be integrated into this model because all eight coping strategies can comprise non-social and social variants. In sum, the CCM offers a parsimonious model that is compatible with and integrates most previous lines of research on stress coping.

## Author contributions

RS: Writing – original draft, Writing – review & editing. HC: Writing – review & editing. FS: Writing – review & editing.
